# Protective effects of in vitro treatment with zinc, d-aspartate and coenzyme q10 on human sperm motility, lipid peroxidation and DNA fragmentation

**DOI:** 10.1186/1477-7827-11-81

**Published:** 2013-08-16

**Authors:** Riccardo Talevi, Vincenza Barbato, Ilaria Fiorentino, Sabrina Braun, Salvatore Longobardi, Roberto Gualtieri

**Affiliations:** 1 Dipartimento di Biologia, Università di Napoli “Federico II”, Complesso Universitario di Monte S Angelo, Via Cinthia, Napoli 80126, Italy; 2 Merck Serono S.p.A., Via Casilina, Roma 125-00176, Italy

**Keywords:** Spermatozoa, Antioxidants, Motility, Lipid peroxidation, DNA fragmentation

## Abstract

**Background:**

Spermatozoa are extremely vulnerable to oxidative stress caused by the unbalance between concentrations of reactive oxygen species and antioxidant scavenging systems present inside the male reproductive tract. In spite of a large number of clinical studies that claimed the beneficial effects of antioxidant oral administration on sperm physiology and fertility, only a few studies were addressed to evaluate their effects on spermatozoa in vitro. Main aims of the present study were to assess the influence of zinc, D-aspartate and coenzyme Q10, included in the dietary supplement Genadis (Merck Serono), on human sperm motility, DNA fragmentation and lipid peroxidation.

**Methods:**

Semen samples, obtained from forty-four patients (23–30 years of age) were enrolled in this study, twenty-four were normospermi**c** and twenty patients were oligospermic. Semen samples were analysed for sperm progressive motility and kinetics through computer assisted analysis, DNA fragmentation and lipid peroxidation.

**Results:**

Main results showed that in both normo and oligospermic samples, total and progressive sperm motility is maintained by in vitro treatment with zinc, D-aspartate and coenzyme Q10, whereas a significant decrease of these parameters occurs in parallel samples incubated in medium alone. Zinc, D-aspartate and coenzyme Q10 also prevented the decrease of sperm kinetics but such an effect was highly significant only in oligospermic samples. Moreover, they also protected spermatozoa by the increase of DNA fragmentation and lipid peroxidation.

**Conclusions:**

Zinc, D-aspartate and coenzyme Q10 exert a direct protective effect on human spermatozoa preventing the decrease of motility and the increase of DNA fragmentation and lipid peroxidation during in vitro culture.

## Background

Oxidative stress manifests when reactive oxygen species (ROS), that include hydroxyl radicals, superoxide anions and hydrogen peroxide [1-3], overwhelm the antioxidant defence system in cells. Spermatozoa were the first cell type showing a potential susceptibility to oxidative damage [4]. They are particularly vulnerable to oxidative stress caused by the imbalance between ROS and antioxidant scavenging systems of the male reproductive tract. In physiological conditions, a balance called oxidative stress status exists between ROS production and antioxidant scavenging system in the male reproductive tract [5] where the principal sources of ROS are leukocytes and abnormal spermatozoa [6,7]. However, low levels of ROS are essential for the regulation of sperm functions such as capacitation, acrosome reaction, and sperm-oocyte fusion [8,9]. Spermatozoa are particularly vulnerable to oxidative stress as they are characterized by: 1) high polyunsaturated fatty acid content; 2) intrinsic deficiencies in intracellular antioxidant enzymes; and 3) limited capacity of DNA repair. The reproductive tract contains a powerful array of enzymatic and non-enzymatic antioxidant molecules (such as vitamins C and E, folate, zinc, selenium, carnitine and carotenoids) that act as scavengers of ROS and protect spermatozoa. High ROS levels have been detected in the semen samples of 25% to 40% of infertile men [10,11]. Moreover, male idiopathic infertility has been correlated with high seminal ROS levels and low antioxidant potential compared to healthy fertile controls [12].

ROS cause infertility by two principal mechanisms damaging the sperm membrane via lipid peroxidation with consequent reduction of sperm motility and ability to fuse with the oocyte [13,14], and sperm DNA, compromising the paternal genomic contribution to the embryo [15-18]. Several studies showed that men with high dietary intake of antioxidants have a lower frequency of sperm aneuploidy and improved semen quality compared with men with low intake [19,20]. As a result, over the last decade, several antioxidant nutraceutical formulations have been developed and proposed as a therapy for male infertility. Recently, a new nutraceutical formulation containing zinc, D-aspartate (D-Asp) and Coenzyme Q10 (CoQ10), a combination of antioxidants and micronutrients, has been developed by Merck Serono.

Zinc is a cofactor for several metalloenzymes involved in DNA transcription and protein synthesis and also has anti-apoptotic and antioxidant properties [21]. Zinc therapy in men with asthenozoospermia resulted in a significant increase in sperm concentration, progressive motility, sperm integrity and improved conception and pregnancy rates [22].

D-Asp is an endogenous amino acid found in the nervous and endocrine system of various animal species. High concentrations of D-Asp have been found in Leydig cells, in rat testis fluid, and in epididymal spermatozoa [23-25]. Moreover, the concentration of D-Asp in seminal plasma and in spermatozoa were significantly reduced in oligoasthenoteratospermic patients [26].

CoQ10 is a component of the mitochondrial respiratory chain, playing a crucial role both in energy metabolism and as liposoluble chain-breaking antioxidant for cell membranes and lipoproteins [27,28]. CoQ10 biosynthesis is markedly active in testis [29], and high levels of its reduced form ubiquinol are present in spermatozoa [30,31], suggesting a protective role as antioxidant. Different studies demonstrated decreased levels of CoQ10 and its reduced form in seminal plasma and spermatozoa of infertile men with idiopathic and varicocele-associated asthenospermia [32]. Moreover, the exogenous administration of CoQ10 has been reported to improve sperm motility [33].

Although several studies evaluated the influence of seminal plasma levels or oral administration of zinc, D-Asp or CoQ10 on semen quality [33-41], only few data are available on their effects in vitro [34,42]. Therefore, it is not clear whether the beneficial effects of the therapy on sperm physiology were due to an improvement of spermatogenesis or also to a direct effect on spermatozoa. Main aims of the present study were to assess the in vitro effects of zinc, D-Asp and CoQ10 on human sperm motility, lipid peroxidation and DNA fragmentation.

## Methods

### Chemicals

Sydney IVF Gamete Buffer was from Cook Medical (Ireland). Zinc chloride, D-aspartic acid, Coenzyme Q10, paraformaldehyde, Triton X100, sodium citrate, Hoechst 33342, thiobarbituric acid (TBA), acetic acid, sodium dodecyl sulphate (SDS) were from Sigma Chemical Company (Milan, Italy). In Situ Cell Death Detection Kit, Fluorescein was from Roche Diagnostics (Milan, Italy). Image It Lipid Peroxidation kit was from Invitrogen (Monza, Italy).

### Patients

This study was approved by our Institutional Review Board. Semen samples were collected, following written informed consent, from 60 patients (23–30 years of age). Samples were analysed according to criteria of World Health Organization (2010) [43] for concentration and percent progressive motility through Sperm Class Analyzer (SCA Microptic S.L. Barcelona, Spain), on a heated stage at 37°C. Semen samples with total and progressive motility below the normal values (n = 16) were excluded from the analysis to avoid confounding variables on the effect of antioxidants on motility maintenance. Twenty-four normospermic and twenty oligospermic patients were included in the study.

Semen samples were washed in Sydney IVF Gamete Buffer at 650 g for 10 min and the pellets were suspended in fresh Sydney IVF Gamete Buffer, divided into aliquots and treated or analysed as detailed below.

### Effects of single molecules on total and progressive sperm motility

**Figure 1:**
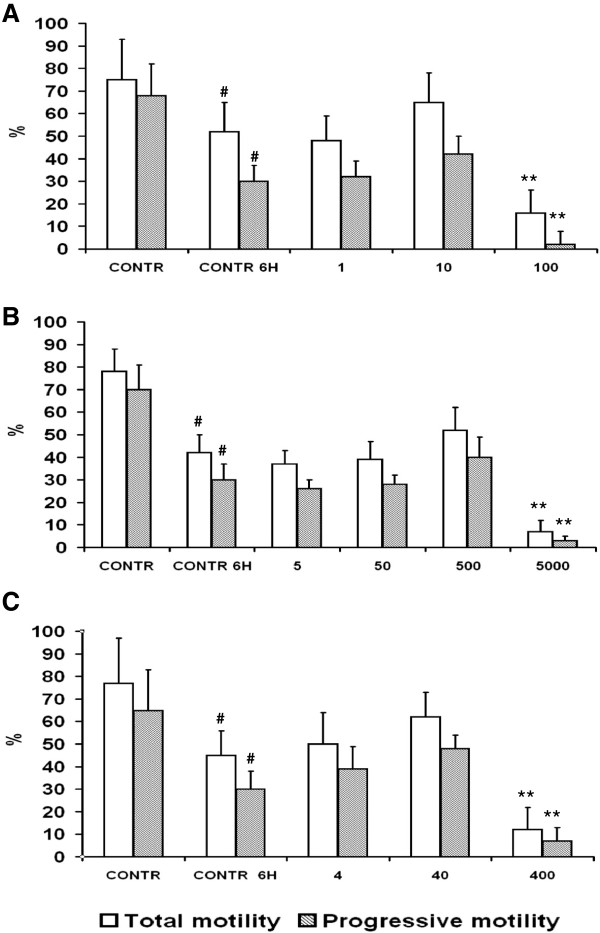
**Effects of single molecules on total and progressive sperm motility.** Total and progressive motility of sperm suspensions (n = 5) treated with different concentrations of **(A)** zinc (1,10,100 μg/ml), **(B)** D-Asp (5,50,500,5000 μg/ml), or **(C)** CoQ10 (4,40,400 μg/ml), at 6 h of incubation. # Significant differences versus control at 0 h (P < 0.05). ** Significant differences versus control at 6 h (P < 0.01).

The following stock solutions were prepared: zinc chloride 10 mg/ml in ethanol, D-Asp 50 mg/ml in gamete buffer, CoQ10 50 mg/ml in chloroform. In all experiments, controls were added with the same concentration of vehicle present in zinc, D-Asp and CoQ10 treated samples. In a preliminary series of experiments, total and progressive motility was assessed on sperm suspensions (n = 5) treated with different concentrations of zinc (1,10,100 μg/ml), D-Asp (5,50,500,5000 μg/ml), or CoQ10 (4,40,400 μg/ml), for 6 h. Samples were loaded into a Makler chamber and analysed on a heated stage at 37°C every hour and until 6 h after treatment, at a Nikon TE 2000 inverted microscope connected to a Basler Vision Technology A312 fc camera with a positive phase contrast 10X objective through Sperm Class Analyzer (SCA Microptic S.L. Barcelona, Spain). For each time point at least 400 cells were acquired and analyzed. Data showed a significant decrease of motility in control samples at 6 h of incubation (Figure 1). At that time, individual optimal concentrations that prevented the decrease of total and progressive motility compared to controls were zinc chloride 10 μg/ml (Figure 1A), D-Asp 500 μg/ml (Figure 1B), CoQ10 40 μg/ml (Figure 1C). Therefore, all experiments mentioned below were performed treating samples in combination, with zinc chloride 10 μg/ml, D-Asp 500 μg/ml, CoQ10 40 μg/ml.

### Effect of combined treatment with antioxidants on sperm motility and kinetics

Spermatozoa of normo (n = 24) and oligospermic patients (n = 20) were evaluated through Sperm Class Analyser for progressive motility and kinetics, i.e. curvilinear velocity (VCL), straight-line velocity (VSL), average path velocity (VAP), with the following software settings: frame rates: 25 frames/s, number of frames: 10 frames/object, velocity limit for slow spermatozoa: 10 μm/s, velocity limit for medium spermatozoa: 15 μm/s, velocity limit for rapid spermatozoa 35 μm/s, minimal linearity 50%, straightness for progressive fast spermatozoa 80%.

### Sperm lipid peroxidation

Experiments addressed to study the influence of zinc, D-Asp and CoQ10 on sperm lipid peroxidation were performed on four responsive normo and four oligospermic samples. As no significant differences were found between normo and oligospermic samples, findings are reported as cumulative data.

Sperm lipid peroxidation was evaluated biochemically through the malondialdehyde (MDA) assay and in situ through labeling with C11-BODIPY^581/591^.

The MDA assay was performed through a protocol first described by Ohkawa et al. [44]. TBA forms colored adduct which were quantified at 532 nm. A 100 μl aliquot of control and treated sperm suspensions at 0 and 6 h after beginning of the treatment with zinc chloride 10 μg/ml, D-Asp 500 μg/ml, CoQ10 40 μg/ml was mixed with 1.5 ml of 20% acetic acid (pH 3.5). Then, 0.5 ml of 8.1% SDS, 1.5 ml of 0.8% TBA and 0.9 ml of phosphate buffer (pH 7.4) were added and the mixture was vortexed. The reaction mixture was incubated in a boiling water bath for 1 h. After cooling at room temperature, 5 ml of butanol:pyrimidine mixture (15:1) was added and the reaction mixture was centrifuged at 4000 g for 20 min. A clear supernatant obtained after centrifugation was used for measuring the absorbance at 532 mm against reagent blank. A series of known concentrations for standard of MDA (1 nM/μl) were also run simultaneously and a standard curve was plotted. The absorbance of samples was plotted against the standard curve and the concentration of MDA was determined as nM of MDA per 10^6^ spermatozoa.

Visualization of lipid peroxidation in situ was carried out through labeling with C11-BODIPY^581/591^, a fatty acid analogue that readily incorporates into cell membranes and whose fluorescence irreversibly changes from red to green upon exposure to ROS [45]. Sperm suspensions were labeled with 10 μM C11-BODIPY^581/591^ in gamete buffer for 30 min at 37°C and then washed twice through centrifugation at 650 g for 10 min and treated with either vehicle (controls) or antioxidants for 6 h. Samples were then analyzed at 37°C at a Leica TCS SP5 confocal microscope (Leica, Milan, Italy) fitted with an Argon (488 nm) laser. Red emission from intact C11-BODIPY^581/591^ was detected at 580–620 nm and green emission that indicated peroxidation at 495–560 nm. Percentages of peroxidized spermatozoa was determined counting at least 200 cells per sample.

### Sperm DNA fragmentation

Experiments addressed to study the influence of zinc, D-Asp and CoQ10 on sperm DNA fragmentation were performed on four responsive normo and four oligospermic samples. As no significant differences were found between normo and oligospermic samples, findings are reported as cumulative data.

Sperm DNA fragmentation was measured by the TUNEL assay in control and treated samples at 0 and 6 h after treatment. Different aliquots of the same semen samples were treated with zinc, D-Asp and CoQ10, or vehicle. Samples were then centrifuged at 400 g for 10 min, fixed in 4% paraformaldehyde in phosphate buffered saline (PBS) for 30 min at room temperature, washed three times in PBS through centrifugation at 400 g for 10 min, smeared on glass slides and air dried. Samples were permeabilized in 0.1% Triton X-100 in 0.1% sodium citrate for 5 min at 4°C, washed in PBS three times for 10 min and then incubated in TUNEL reaction mixture according to the manufacturer for 1 h in the dark at 37°C . At the end of incubation, slides were washed in PBS as above, labelled with Hoechst 33342 10 μg/ml for 7 min at room temperature, washed again in PBS, mounted and observed at a Nikon TE 2000 fluorescence microscope. Images were acquired using a Nikon DS-cooled camera head DS-5Mc connected to a Nikon DS camera control unit DS-L1 using the same exposure conditions. In each procedure negative and positive controls were prepared by omission of TdT in the reaction mixture or by pretreatment with 1 mg/ml DNase I (Roche Diagnostics) for 10 min at room temperature. At least 200 spermatozoa were analysed in each sample.

### Statistical analysis

The data are presented as the mean ± SD. Overall, analysis was performed by Fisher’s exact test or by the estimate model of ANOVA followed by the Tukey’s honestly significant difference test for pairwise comparisons when overall significance was detected.

## Results

### Sperm progressive motility and kinetics

**Figure 2:**
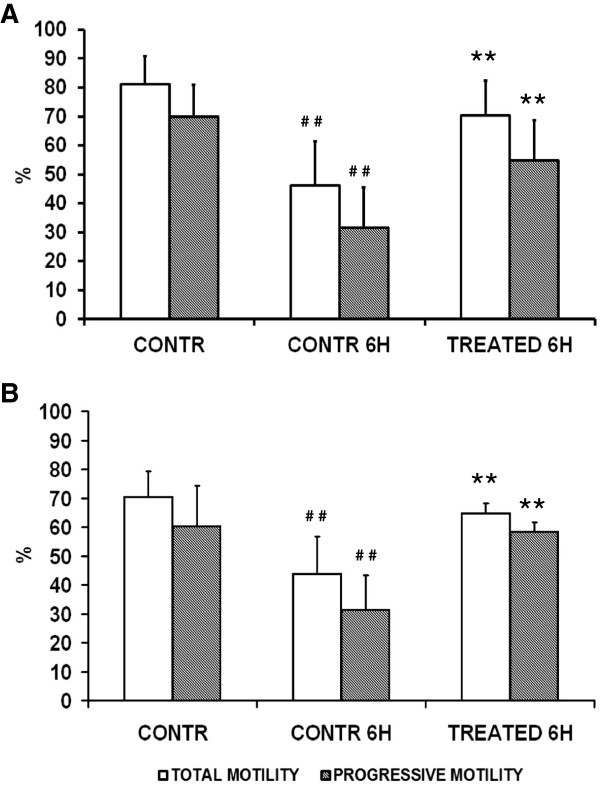
**Effects of zinc, D-Asp and CoQ10 on total and progressive sperm motility.** Normospermic (n = 18) **(A)** and oligospermic (n = 15) **(B)** sperm suspensions treated with zinc, D-Asp and CoQ10. # # Significant differences versus control at 0 h (P < 0.01); ** Significant differences versus control at 6 h (P < 0.01).

To understand whether zinc, D-Asp and CoQ10 affect progressive motility, spermatozoa of normo (n = 24) and oligospermic patients (n = 20) were treated with zinc chloride 10 μg/ml, D-Asp 500 μg/ml, CoQ10 40 μg/ml. Data showed that a positive response was observed in all sperm suspensions in which the control had a significant decrease of motility during the 6 h of analysis (75% of patients: 18 normo and 15 oligospermic). In responsive normospermic patients (Figure 2A) initial total motility was 81 ± 9.6%, and progressive motility was 70 ± 10.9%. A significant decrease of both values was observed after 6 h of incubation in medium alone (motility 46 ± 15.2%, progressive motility 31.5 ± 13.9%; control 0 h vs control 6 h, P < 0.01). Interestingly, treatment with zinc, D-Asp and CoQ10 prevented the drop of motility observed in parallel control samples (motility 70.2 ± 12%, progressive motility 54.8 ± 13.9%; 6 h, control vs treatment, P < 0.01).

In responsive oligospermic patients (Figure 2B) initial total motility was 70.3 ± 9%, and progressive motility was 60.3 ± 14%. A significant decrease of both values was observed after 6 h of incubation in medium alone (motility 43.6 ± 13%, progressive motility 31.3 ± 12%; control 0 h vs control 6 h, P < 0.01). Interestingly, treatment with zinc, D-Asp and CoQ10 prevented the drop of motility observed in parallel control samples (motility 64.6 ± 3.5%, progressive motility 58.3 ± 3.2%; 6 h, control vs treatment, P < 0.01).

**Figure 3:**
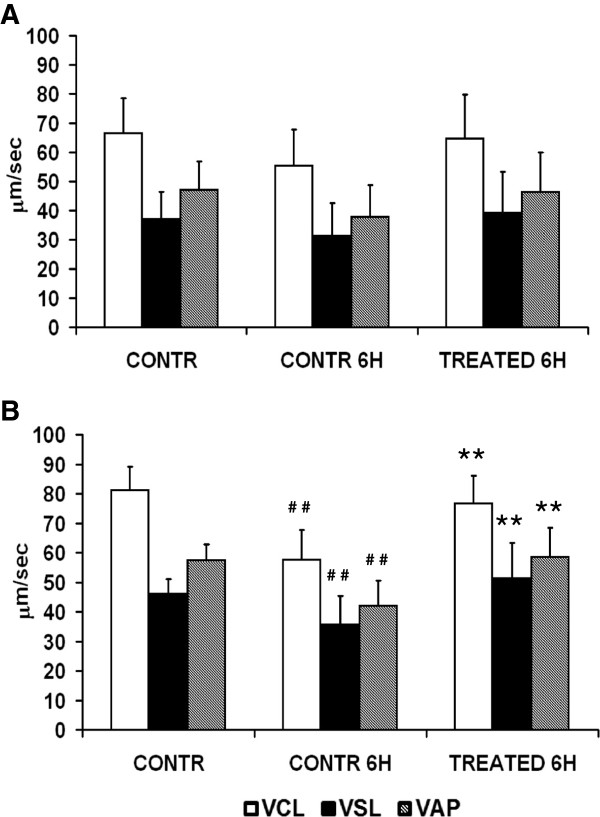
**Effects of zinc, D-Asp and CoQ10 on sperm kinetics.** Normospermic ( n = 18) **(A)** and oligospermic (n = 15) **(B)** sperm suspensions. VAP, path velocity; VCL, curvilinear velocity; VSL, straight line velocity. # # Significant differences versus control at 0 h (P <0.01); ** Significant differences versus control at 6 h (P < 0.01).

Initial mean kinetic parameters in responsive normospermic patients (Figure 3A) were: VCL 66.6 ± 11.8 μm/sec, VSL 37 ± 9.49 μm/sec, VAP 47.1 ± 9.6 μm/sec. After 6 h of incubation, all values decreased in control samples (VCL 55.5 ± 12.3 μm/sec, VSL 31,4 ± 11.1 μm/sec, VAP 37.9 ± 10.8 μm/sec; control 0 h vs control 6 h, not significant) whereas they were maintained in treated parallel samples (VCL 64.8 ± 14.8 μm/sec, VSL 39,3 ± 13.8 μm/sec, VAP 46.4 ± 13.6 μm/sec; 6 h, control vs treatment, not significant). Although a similar trend was observed in responsive oligospermic patients (Figure 3B), the drop of kinetics at 6 h in medium alone was highly significant (control 0 h vs control 6 h, VCL 81.1 ± 8 vs 57.7 ± 10 μm/sec, VSL 46 ± 5 vs 35.7 ± 9.6 μm/sec, VAP 57.4 ± 5.4 vs 42.1 ± 8.4 μm/sec, P < 0.01) and it was prevented by the treatment with zinc, D-Asp and CoQ10 (VCL 76.6 ± 9.4 μm/sec, VSL 51,3 ± 11.9 μm/sec, VAP 58.5 ± 9.9 μm/sec; 6 h, control vs treatment, P < 0.01).

### Sperm lipid peroxidation and DNA fragmentation

**Figure 4:**
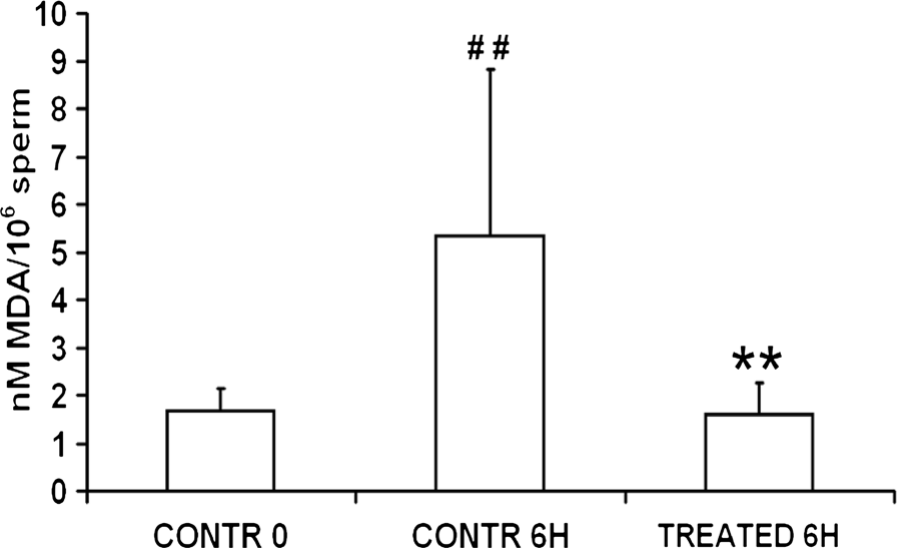
**Effects of zinc, D-Asp and CoQ10 on sperm lipid peroxidation.** Malondialdehyde assay of normospermic (n = 4) and oligospermic (n = 4) sperm suspensions (pooled data). # # Significant differences versus control at 0 h (P <0.01); ** Significant differences versus control at 6 h (P < 0.01).

The lipid peroxide contents, as determined by MDA assay (Figure 4), in sperm samples at 0 h was 1,67 ± 0,47 nM MDA/10^6^ spermatozoa. These initial values were markedly and significantly increased after 6 h of incubation in medium alone (5,35 ± 3,48 nM MDA/10^6^ spermatozoa, control 0 h vs control 6 h, P < 0.01) whereas lipid peroxidation was prevented by the treatment with zinc, D-Asp and CoQ10 (1,60 ± 0,67 nM MDA/10^6^ spermatozoa; 6 h, control vs treatment, P < 0.01).

**Figure 5:**
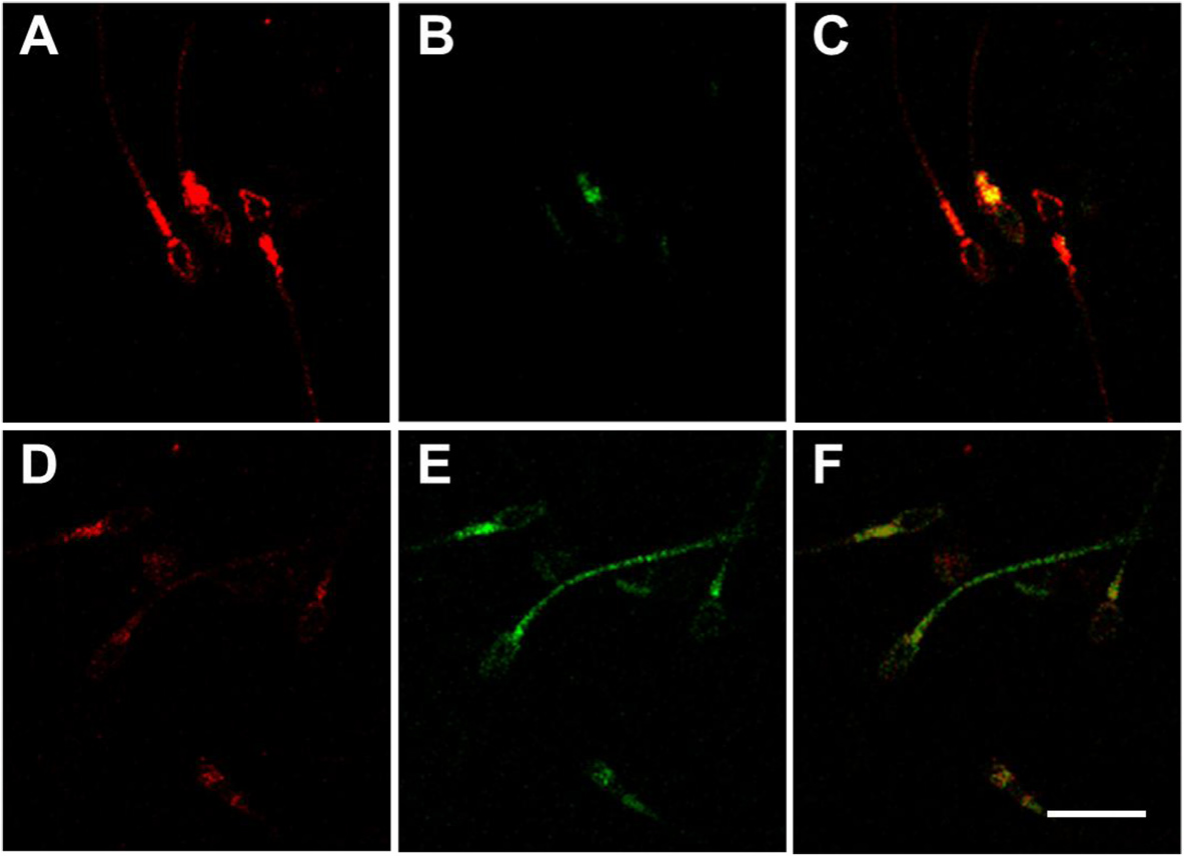
**Confocal laser scanning micrographs of sperm lipid peroxidation.** Spermatozoa labeled with C11-BODIPY^581/591^ to visualize lipid peroxidation. **A-C**, unperoxidized spermatozoa; **D-F** peroxidized spermatozoa. **A**,**D**, red channel; **B**,**E**, green channel; **C**,**F**, merge. Bar, 10 μm.

**Figure 6:**
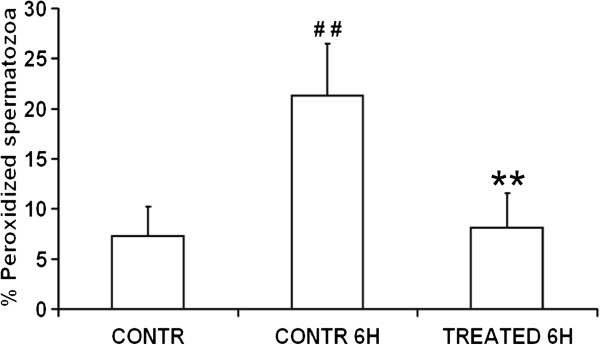
**Effects of zinc, D-Asp and CoQ10 on sperm lipid peroxidation.** Sperm suspensions of normospermic (n = 4) and oligospermic (n = 4) samples labeled with C11-BODIPY^581/591^ (pooled data). # # Significant differences versus control at 0 h (P < 0.01); ** Significant differences versus control at 6 h (P < 0.01).

Spermatozoa were washed and labeled with the fluorescent lipid peroxidation reporter probe C11-BODIPY^581/591^ and then treated for 6 h with zinc, D-Asp and CoQ10 or in medium alone. At the confocal laser scanning microscope, unperoxidized spermatozoa were characterized by a red fluorescence localized over the whole cell (Figure 5A) whereas peroxidized spermatozoa were characterized by a green fluorescence mainly at the level of the midpiece and tail but in some cases distributed also on the sperm head plasma membrane (Figure 5E). Most spermatozoa in the initial suspensions and after 6 h of treatment were unperoxidized (Figure 5A), whereas in control samples at 6 h there was a decrease of spermatozoa with red fluorescence and a concomitant increase of cells showing a green fluorescence (Figure 5D,E). The percentage of peroxidized spermatozoa (Figure 6) after 6 h of incubation was increased three times compared to the initial sperm suspensions (21.3 ± 5.2 vs 7.3 ±5.2, P < 0.01) whereas such an increase was prevented in treated samples (6 h, control vs treatment, 21.3 ± 5.2 vs 8.1 ± 3.5, P < 0.01).

**Figure 7:**
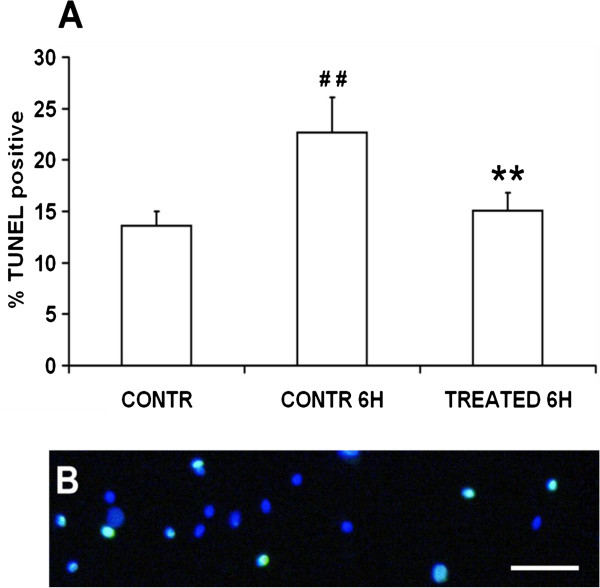
**Effects of zinc, D-Asp and CoQ10 on sperm DNA fragmentation. A)** Sperm suspensions of normospermic (n = 4) and oligospermic (n = 4) samples labeled through the TUNEL assay (pooled data). **B)** Representative micrograph of spermatozoa labeled with Hoechst (blue) and TUNEL (green). Bar, 20 μm # # Significant differences versus control at 0 h (P < 0.01); ** Significant differences versus control at 6 h (P < 0.01).

Experiments were addressed to evaluate the DNA fragmentation in the initial suspensions and after 6 h of incubation. Data (Figure 7A) showed that the percentage of spermatozoa with fragmented DNA (Figure 7B) significantly increased from 13.6 ± 1.4 in the initial suspensions to 22.7 ± 3.4% after 6 h of incubation (P < 0.01), whereas treatment with zinc, D-Asp and CoQ10 had a protective effect on sperm DNA fragmentation (15 ±1.7%; treatment 6 h vs control 0 h, NS; treatment 6 h vs control 6 h, P < 0.01).

## Discussion

Reactive oxygen species are involved in several cellular signaling mechanisms and they can interact with lipids, proteins and DNA, leading to severe pathologic conditions. High ROS levels are detrimental to gametes, and compromise their function through lipid peroxidation, protein damage and DNA strand breakage [18]. Although, spermatozoa physiologically produce ROS promoting sperm capacitation, in some pathological conditions the semen ROS levels exceeds the sperm antioxidant defenses and lead to a state of oxidative stress that could impair not only fertilization but also embryo development [18]. In vivo studies suggested that oral administration of antioxidants improves semen quality and pregnancy rates in subfertile men [46,47]. However, among several studies on the effect of antioxidants on semen quality, only a few were addressed to understand their direct action on sperm physiology in vitro. To this end, in the present study we evaluated the effects of zinc, D-Asp and CoQ10 on sperm physiology in vitro. Main results demonstrated that the in vitro treatment of human spermatozoa with zinc, D-Asp and CoQ10 1) preserves sperm motility and kinetics; 2) avoids sperm lipid peroxidation, and 3) DNA fragmentation.

To our knowledge, the present work represents the first addressing the effects of a combined in vitro treatment with zinc, D-Asp and CoQ10 on human spermatozoa. Several studies have been carried out on the role played by each of these molecules on spermatogenesis, sperm quality and fertility.

Zinc concentration in seminal plasma is generally higher than in serum and has been positively correlated with sperm count and motility [21,48,49]. On the other hand, a negative effect of high zinc levels in seminal plasma or in sperm tails on sperm motility has been reported by others [50,51]. Despite these contradictory results, the role of zinc as an antioxidant is well established. Zinc in vitro is able to inhibit both superoxide anion generation and SOD-like activity in spermatozoa of infertile men [52].

Little is known about the role of D-Asp in human reproduction. The concentration of D-Asp in seminal plasma and in spermatozoa was significantly reduced in oligoasthenoteratospermic men [26]. Moreover, DL-Aspartic acid administration has been reported to improve semen quality in rabbits [37].

CoQ10 has a bioenergetic and an antioxidant role and has been suggested to be involved in male infertility [53]. High levels of oxidative stress and a low antioxidant capacity in varicocele patients has been correlated to an altered distribution of CoQ10 in spermatozoa and seminal plasma [32]. CoQ10 administration has been reported to have a positive role in the treatment of asthenozoospermia [33].

Our results on the in vitro effects of zinc, D Asp and CoQ10 confirm the role played by the single molecules on human spermatozoa and demonstrate that they are able to protect spermatozoa from the oxidative stress during in vitro manipulation. Analysis of sperm motility and kinetics demonstrated that the supplementation of culture media with the three molecules prevents the drop of these values observed in medium alone. Such an effect was evident in 75% of samples in which a significant decrease of motility and kinetics was observed at 6 hours of incubation in medium alone. This indicate that a preliminary analysis of sperm motility dynamics on washed semen samples could help to identify responsive patients. The lack of response in 25% of samples analysed could be due to the presence of a correct balance between antioxidant defenses and ROS generation in those ejaculates.

Sperm plasma membrane plays fundamental roles during sperm transport within the female reproductive tract, in sperm capacitation, in sperm–egg interaction and, finally, in fertilization. For these reasons, the plasma membrane lipid composition of spermatozoa is different from somatic cells for the high content of highly polyunsaturated fatty acids. These unsaturated fatty acids confer to the sperm membrane a great fluidity needed to participate in the membrane fusion events associated with capacitation and fertilization. On the other hand this makes spermatozoa particularly vulnerable to the attack by ROS, and therefore more susceptible to undergo lipid peroxidation [54]. Several studies examined the role of in vitro and in vivo antioxidant supplementation in protecting sperm from lipid peroxidation due to an imbalanced ROS production [55-57]. In the present paper, data demonstrated that treatment with zinc, D Asp and CoQ10 protects sperm plasma membrane from lipid peroxidation. This protection could be due to CoQ10 that has been previously reported to decrease lipid peroxidation when administered in a rat model of ischemia/reperfusion injury [41].

High levels of ROS can induce DNA damage in spermatozoa in every moment of their life. This damage can be produced during comigration of mature and immature spermatozoa from the seminiferous tubules to the caudal epididymis where sperm are highly packed and this would facilitate ROS-induced DNA damage [58]. Moreover, the process of DNA fragmentation in spermatozoa progresses even after ejaculation. In vitro incubation of swim-up selected human spermatozoa results in a progressive increase in the percentage of DNA fragmented sperm [59].

In agreement with those studies we found that sperm culture for 6 hours induces an increase of sperm DNA fragmentation and, more interestingly, it is prevented by antioxidant treatment. According to Aitken et al., [18] sperm oxidative stress not only could impair the sperm fertilizing ability but also its competence to sustain a correct embryo development. Moreover, DNA damage in human spermatozoa has been correlated with increased miscarriage rates and morbidity in the offspring [59,60]. The possibility to answer these questions directly in the human is limited by a number of ethical and methodological reasons. Studies in animal models could provide insights into these fundamental questions.

## Conclusions

Overall, data here presented demonstrate that in vitro treatment of human spermatozoa with zinc, D Asp and CoQ10 exerts a direct protective effect on sperm motility, kinetics, lipid peroxidation and DNA fragmentation during handling and extended culture. As semen processing, handling and cryopreservation represent crucial steps during assisted reproduction techniques, the supplementation of new sperm culture media with zinc, D Asp and CoQ10 could be an useful tool to preserve sperm physiology against damage induced by oxidative stress.

## Abbreviations

CoQ10: Coenzyme Q10; D-Asp: D-aspartate; MDA: Malondialdehyde; PBS: Phosphate buffered saline; ROS: Reactive oxygen species; SCA: Sperm Class analyser; SDS: Sodium dodecyl sulphate; TBA: Thio barbituric acid; VAP: Average path velocity; VCL: Curvilinear velocity; VSL: Straight-line velocity.

## Competing interests

BV, FI, BS, TR and GR have no competing interests to disclose. LS is employed by Merck Serono S.p.A., Rome, Italy.

## Authors’ contributions

BV, FI and BS participated in sperm progressive motility and kinetics, lipid peroxidation and DNA fragmentation analysis. LS participated in the design of the study and research ethics application. TR and GR were the principal investigators, conceived the study prepared the manuscript. All authors read and approved the final manuscript.
